# Impairment-driven cancer rehabilitation in patients with neoplastic spinal cord compression using minimally invasive spine stabilization

**DOI:** 10.1186/s12957-020-01964-y

**Published:** 2020-07-25

**Authors:** Yukako Ishida, Hideki Shigematsu, Shinji Tsukamoto, Yasuhiko Morimoto, Eiichiro Iwata, Akinori Okuda, Sachiko Kawasaki, Masato Tanaka, Hiromasa Fujii, Yasuhito Tanaka, Akira Kido

**Affiliations:** 1 Department of Rehabilitation Medicine, Nara Medical University, 840 Shijo-cho, Kashihara, Nara, 634-8522 Japan; 2 Department of Orthopedic Surgery, Nara Medical University, Nara, Japan

**Keywords:** Impairment, Cancer rehabilitation, Percutaneous pedicle screws, Spinal metastasis, Minimally invasive spine stabilization

## Abstract

**Background:**

Neoplastic spinal cord compression is a cause of severe disability in cancer patients. To prevent irreversible paraplegia, a structured strategy is required to address the various impairments present in cancer patients. In this study, we aimed to identify the status where rehabilitation with minimally invasive spine stabilization (MISt) effectively improves ADL.

**Methods:**

We retrospectively reviewed 27 consecutive patients with neoplastic spinal compression who were treated with MISt. We classified the impairments of patients through our multidisciplinary tumor board based on spine-specific factors, skeletal instability, and tumor growth. The neurological deficits, progress of pathological fracture, incidence of vertebral collapse, and postoperative implant failure were examined. Changes of the Barthel index (BI) scores before and after surgery were investigated throughout the clinical courses.

**Results:**

The average duration to ambulation was 7.19 ± 11 days, and we observed no collapse or progression of paralysis except in four cases of complete motor paraplegia before the surgery. Neurological deficiency was improved to or maintained at Frankel’s grade E in 16 patients, remained unchanged in 6 patients (in grades B, C, D), and worsened in 5 patients. BI score comparisons before and after surgery in all patients showed statistically significant increments (*p* < 0.05). On further analysis, we noted good functional prognosis in patients capable of ambulation within 7 days (*p* < 0.05) and in patients who could survive longer than 3 months after the surgery (*p* < 0.05).

**Conclusions:**

In various cancer patients with neoplastic spinal cord compression, skeletal instability as the primary impairment is a good indication for MISt, as the patients showed early ambulation with improved BI scores.

## Background

Progress in early cancer detection and effective treatment has rapidly increased the number of cancer survivors. In the US, the 5-year survival rate for all cancers has reached 69.2% for men and 69.1% for women, and these are expected to increase in future years [1]. In Japan, the 5-year survival rate for all cancers has reached 62% for men and 66.9% for women [2]. Accordingly, the demand for cancer rehabilitation is increasing. Because of the long time periods associated with cancer treatment, most survivors do not return to their previous state of well-being [3, 4].

According to the International Classification of Impairments, Disabilities, and Handicaps (ICIDH) developed by the World Health Organization, impairment refers to a problem associated with a bodily structure or organ, whereas disability refers to a functional limitation in a particular activity [5]. The ICIDH was substituted by the International Classification of Functioning, Disability, and Health (ICF) in 2001 [6], which combines more positive aspects of body functions, activities, and participation. Cancer patients suffer from a wide range of impairments, partially because of either the disease itself or the adverse effects of treatment. Both physical and psychological impairments contribute to decreased quality of life which may result in disability [5]. Recently, many researchers have demonstrated that cancer rehabilitation improves physical and cognitive impairments, societal participation, and QOL at every stage along the treatment course for a variety of cancer type [7–10].

Impairment-driven cancer rehabilitation is a model advocated by Silver et al. since 2013 [11]. They emphasized the importance of identifying physical impairments because disability is frequently driven by the interactions of multiple physical impairments. They stressed that an important component of the rehabilitation care continuum should be offered to survivors only after their impairments have been identified and treated optimally, and safety precautions and contraindications have been identified and documented. To improve the oncology-rehabilitation interface, screening and treating for all impairments should be performed throughout the course of care [11].

Among the different impairments in cancer patients, neoplastic spinal compression is unique because the severity of impairment (or of disability) does not correlate with the pathological grade of malignancy. The impairment is associated with the level of spinal cord injury and the volume of spinal compression [12–14]. Thus, we assume that disability from neoplastic spinal compression can be prevented with appropriate surgical, medical, and rehabilitative interventions aimed to treat these spine-specific factors and such interventions would be effective even in patients at the palliative stage.

We evaluated the spine-specific factors based on two independent biological aspects: skeletal instability and tumor growth [15, 16]. Skeletal instability is caused by the destruction of bony structures. A pathological fracture may occur and fragments in the spinal canal may cause neurological deficit. In terms of tumor growth, the volume of rapidly-growing tumors in the spinal canal may threaten the spinal cord directly, even if skeletal stability was preserved. Regarding surgical approaches, wide resection has been considered the curative treatment because both local and marginal resection may result in higher rates of recurrence. Therefore, conservative surgeries for local control have rarely been performed for these patients [17, 18]. However, to treat skeletal instability, some surgical approaches have been reported [19–22].

Minimally invasive spine stabilization (MISt) with percutaneous pedicle screws (PPS) have shown advantages of relatively lower blood loss, less morbidity, shorter hospital stays, lower postoperative infections and immediate mobilization without the need for external bracing [20–22]. Several studies have reported improved activities of daily living (ADL) after MISt with PPS as a palliative surgery for patients with neoplastic spinal cord compression [20–22]. However, since most studies lack the impairment-driven approach, surgical indications still remain unclear.

In this study, we hypothesized that there are certain cancer populations, whose impairments are suitable for rehabilitation with MISt. For these patients, this intervention may improve ADL more effectively than for other cancer patients. This study aimed to identify the status at which rehabilitation with MISt effectively improves ADL.

## Methods

We retrospectively reviewed the data of 27 consecutive patients with neoplastic spinal compression who were treated with MISt and received immediate rehabilitation thereafter. Before the surgery, the status of impairment was evaluated based on both spine-specific factors and other factors. The neurological deficit, ambulation status, progress of pathological fracture, collapse, and postoperative implant failure were examined. Furthermore, the relationship between the improvement of Barthel index (BI) [23] and prognosis was statistically analyzed.

### Patients

This retrospective study was conducted at Nara Medical University Hospital. The study protocol was approved by the institutional review board of the hospital and was conducted in concordance with principles of the Declaration of Helsinki and with the laws and regulations of Japan. A consecutive cohort of 27 patients with neoplastic spinal cord compression from 2014 to 2017 who met the surgical indications described below were enrolled. The treatment strategy for all the patients was assessed by the multidisciplinary tumor board of our hospital. Informed consent was obtained through our website. Inclusion criterion was patients with MISt for neoplastic spinal cord compression during the study period. We provided informed consent to all patients and no cases opted-out. No cases were excluded from the study. Follow-up periods averaged at 420 ± 357 days (range, 30–1305 days).

### Surgical indication for MISt

Surgical indication for MISt was assessed comprehensively by the multidisciplinary tumor board for skeletal metastasis, based on clinical findings including (1) spinal instability, (2) radiological spinal compression, (3) prognosis, (4) feasibility for the stabilization surgery (whether patients had multiple spinal lesions), (5) presence of pain or neurological deficits. Spinal instability was evaluated using the spinal instability neoplastic scale (SINS) score [15]. The SINS is generated by tallying each score from the 6 individual components (location, pain, bone lesion quality, spinal alignment, vertebral body collapse, and posterolateral involvement of spinal elements). It has excellent inter- and intraobserver reliability in determining three clinically relevant categories of stability [15]. Score ≥ 7 was classified as potentially unstable or unstable. To assess the degree of cord compression, the 6-point epidural spinal cord compression (ESCC) grading scale was used [16]. It is a magnetic resonance (MR) imaging-based grading scale, which is based on the degree of impingement of cerebrospinal fluid (CSF) space. The inter- and intraobserver reliability was reported from good to excellent [16]. After grading, neurological findings were evaluated. Regarding prognosis, we referred to the Revised Tokuhashi score [24] and the new Katagiri score [25]. Patients’ with estimated life expectancy of ≥ 1 month were assessed for surgery. The surgery involved a short posterior fixation with PPS placed at two vertebral levels above and two level below the lesion. Patients with multiple spinal lesions expanding the vertebra of planned fixation level were deemed unfeasible for surgery. In addition to fixation, posterior decompression was performed in cases of tumors occupying only the posterior epidural space and was not considered hemorrhagic by radiological and pathological findings.

### Multidisciplinary tumor board for skeletal metastasis

The multidisciplinary tumor board (MDTB) for skeletal metastasis at Nara Medical University Hospital was established in 2010. Since then, the disability/impairment statuses of each patient have been evaluated. The treatment plans for approximately 100 patients have been discussed per year. The monthly board meetings are attended by physicians, medical oncologists, radiation oncologists, diagnostic radiologists, physiatrists, orthopedic oncologists, spine surgeons, advanced practitioners, oncological nurses, and clinical support staffs. Besides the regular monthly board meetings, web discussions are held for emergency cases selected based on the electronic medical record system of the hospital. Cases eligible for presentation include new or existing outpatients or inpatients with skeletal metastasis. The multidisciplinary tumor board supported coordination, communication, and decision-making between team members. Based on these discussions at the board, all patients would immediately receive intensive and regular adjuvant treatments including radiation therapy, chemotherapy, palliative care, and rehabilitation.

### Rehabilitation

Rehabilitation was started 1 day after surgery and involved tasks like sitting, standing, and walking, similar to rehabilitation programs after general (non-oncological) spine surgeries. Four patients, who had complete motor paraplegia before the surgery, did not show any improvement in physical function, yet the stabilized spine enabled them to train in wheelchair riding with decreased pain.

### Outcome evaluation

All patients were hospitalized for surgery. Preoperative measurement was performed at admission, and postoperative measurement was performed at discharge, by medical doctors in the department of rehabilitation medicine. Primary outcome was the Barthel index. Secondary outcomes were neurological deficits using the Frankel Scale (A–E) [26], duration to the start of ambulation exercises, the progress of pathological fractures, incidence of vertebral collapse, postoperative implant failure, and overall survival.

### The Frankel Scale

The Frankel Scale classifies the extent of the neurological/functional deficit into five grades. Frankel grade A patients are those with complete motor and sensory lesions. Grade B patients have sensory only functions below the level of injury. Grade C patients have some degree of motor and sensory function, but their retained/recovered motor function is useless. Grade D patients have useful, but abnormal, motor function below the level of injury. And grade E patients have complete motor/sensory recovery [26].

### Duration to the start of ambulation exercises

Duration (days) to the start of ambulation exercise after the surgery was determined from the medical records.

### Progress of pathological fractures and incidence of vertebral collapse

The progress of pathological fracture, vertebral collapse, or implant failure was checked using routine radiographs after surgery, which were taken every 3–4 weeks.

### Overall survival

Overall survival was assessed at the final follow-up in the outpatient department. Patients were evaluated as alive with disease (AWD) or dead of disease (DOD). We found no patients with no evidence of disease (NED) or continuously disease free (CDF).

### The Barthel index

The Barthel index (BI) is one of the most widely used rating scales for the measurement of activity limitations in patients with neuromuscular and musculoskeletal conditions [23]. It consists of 10 items that measure a person’s daily functioning, including feeding, bathing, grooming, dressing, toilet uses, transfers, mobility, and stair use [23]. BI received high mark reliability and validity ratings in various reports [27–29].

### Statistical analysis

Statistical analysis was performed using the JMP14.0 software (SAS Institute, Cary, NC, USA) and G*Power software 3.1 (University of Dusseldorf). A *p* value of < 0.05 was considered statistically significant. The Wilcoxon signed-rank test was used to evaluate differences in the BI scores before and after surgery because the data was nonparametric using the Shapiro-Wilk test. The Mann-Whitney *U* test, Kruskal-Wallis test, and Spearman correlation test were used to evaluate the association between BI score gain and the variables including tumor progression, ESCC grade (1, 2 versus 3), SINs, and radiation therapy status.

## Results

### Patients’ characteristics

Table 1 shows the patients’ characteristics. Patients included 16 men and 11 women; the mean age at surgery was 65.1 years (range, 18–84 years). Location of primary lesion was lung in 29.6%, liver (or gallbladder) in 14.8%, colon in 11.1%, prostate in 11.1%, kidney in 11.1%, breast in 11.1%, stomach in 3.7%, bone in 3.7%, and lymph node in 3.7%. Frankel’s classification of preoperative paralysis was B in 14.8%, C in 11.1%, D in 11.1%, and E in 63%. Degree of cord compression assessed with the ESCC grading scale included 3.7% with grade 1a, 3.7% with grade 1b, 3.7% with grade 1c, 25.9% with grade 2, and 63.0% with grade 3.

**Table 1:** Patients characteristics (*n* = 27)

Valuable	Value
Gender, *n* (%)	
Male	16 (59.3)
Female	11 (40.7)
Age (mean ± SD), years	65.1 ± 15.665
Range, years	18–84
Primary tumor, *n* (%)	
Lung	8 (29.6)
Liver, gallbladder	4 (14.8)
Colon	3 (11.1)
Prostate	3 (11.1)
Kidney	3 (11.1)
Breast	3 (11.1)
Stomach	1 (3.7)
Bone	1 (3.7)
Lymph node	1 (3.7)
Frankel classification (preoperative), *n* (%)	
A	0 (0)
B	4 (14.8)
C	3 (11.1)
D	3 (11.1)
E	17 (63)
ESCC grade, *n* (%)	
1a	1 (3.7)
1b	1 (3.7)
1c	1 (3.7)
2	7 (25.9)
3	17 (63)

### Duration to ambulation and functional prognosis after surgery

Table 2 shows the clinicopathological data and functional prognosis, including days to ambulation recovery. Although the spinal cord was highly compressed in almost 90% of patients (25.9% of patients had ESCC grade 2 and 63.0% of patients had grade 3), the mean duration to ambulation was 7.19 ± 11 days (range, 1–49 days). Considering the high rate of patients with severe cord compression, it is noteworthy that all patients achieved ambulation after surgery, except for 14.8% of patients who had complete motor paraplegia before surgery. There was no case of implant failure or vertebral collapse after surgery.

**Table 2:** Clinicopathological data and functional prognosis including first ambulation (days) after the surgery

Patient no.	Tumor progression classified by Katagiri et al.	ESCC grade	SINs	Frankel classification, preoperative/postoperative	Pathology level	Instrumentation level	First ambulation training (days after the surgery)	Implant failure	Collapse	RT	BI gain
1	Slow	2	11	D/D	T3	T1-5	2	-	-	+	80
2	Slow	2	10	E/E	L3	L1-5	2	-	-	+	95
3	Slow	3	14	E/D	T3-6, 8, 11, L2	T11-L3	4	-	-	+	90
4	Slow	3	16	E/E	T5.11	T2-L2	7	-	-	+	5
5^*^	Slow	3	11	B/B	T6	T4-8	6	-	-	+	20
6	Slow	3	12	C/D	T4	T2-6	1	-	-	-	25
7	Slow	3	10	E/E	T7	T5-9	2	-	-	+	35
8	Moderate	1a	5	E/E	T7	T5-9	3	-	-	+	0
9	Moderate	1b	11	E/E	T12	T10-L2	2	-	-	+	30
10	Moderate	2	6	E/E	T7	T5-9	4	-	-	+	− 5
11	Moderate	3	16	C/C	T11, L2	T9-L4	4	-	-	+	40
12	Moderate	3	7	E/E	L2	T12-L4	4	-	-	+	0
13	Moderate	3	10	E/C	T8	T5-T11	24	-	-	-	10
14^*^	Moderate	3	8	B/B	T7	T4-10	2	-	-	-	25
15	Rapid	1c	11	C/C	L4, 5	L1-S1	2	-	-	-	50
16^*^	Rapid	2	9	B/C	T6, 8	T4-T10	10	-	-	+	− 15
17	Rapid	2	11	D/D	T9	T7-11	4	-	-	+	60
18	Rapid	2	8	E/E	L3	L1-L5	1	-	-	-	− 10
19	Rapid	2	10	E/D	L4	L2-S1	1	-	-	-	60
20	Rapid	3	11	D/B	T2	T1-3	49	-	-	+	0
21	Rapid	3	11	E/E	T10	T8-12	1	-	-	+	20
22	Rapid	3	13	E/E	T12	T10-L2	NE^**^	-	-	-	− 10
23	Rapid	3	10	E/E	L2	T12-L4	9	-	-	+	− 5
24	Rapid	3	11	E/E	T12	T10-L2	2	-	-	+	95
25	Rapid	3	12	E/B	T6	T3-9	35	-	-	-	− 35
26	Rapid	3	8	E/E	T4, 5	T2-7	1	-	-	+	0
27^*^	Rapid	3	12	B/C	T3	T1-5	5	-	-	-	35

^*^Patients 5, 14, 16, and 27 were nonambulatory due to motor paralysis before the surgery

^**^Patient 22 could not receive the training due to worsening of general condition

### Neurological recovery on the Frankel scale

Table 3 shows the neurological recovery in terms of the Frankel scale. Neurological deficiency was improved to or maintained at grade E in 59.3% of patients, remained unchanged in 22.2% of patients (in grades B, C, D) and worsened in 18.5% of patients.

**Table 3:** Neurological recovery on the Frankel scale

Frankel classification	Number of cases before surgery, *n* (%)	Number of cases after surgery, *n* (%)				
		A	B	C	D	E
A	0 (0)	0 (0)	0 (0)	0 (0)	0 (0)	0 (0)
B	4 (14.8)	0 (0)	2 (7.4)	2 (7.4)	0 (0)	0 (0)
C	3 (11.1)	0 (0)	0 (0)	2 (7.4)	1 (3.7)	0 (0)
D	3 (11.1)	0 (0)	1 (3.7)	0 (0)	2 (7.4)	0 (0)
E	17 (63)	0 (0)	1 (3.7)	1 (3.7)	2 (7.4)	13 (48.1)

### Change of the Barthel index (BI) before and after surgery

**Fig. 1:**
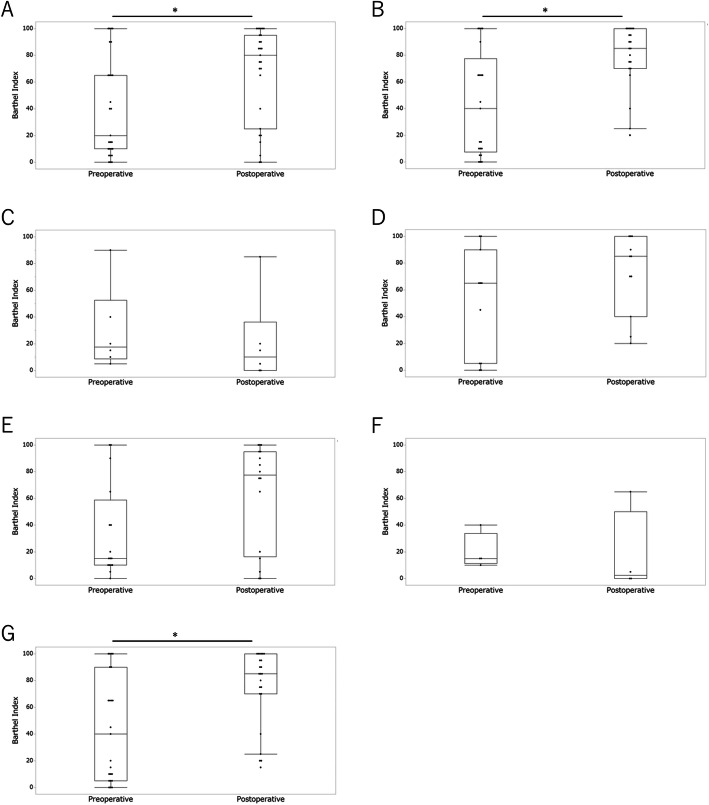
Changes in Figure 1 - small text & poor quality image/text ; Figure 2 - poor quality image/text. Please provide replacement of figure files. Otherwise kindly confirm if we can retain the current presentation.the BI scores after surgery in patients. **a** Comparison in all patients before and after surgery. The 95% confidential interval (C.I.) was 25.4–55.0 in the preoperative and 53.0–80.2 in the postoperative patients; *p* = 0.017. **b** Changes in the BI scores in patients capable of ambulation within 7 days. The 95% C.I. was 25.3–60.8 in the preoperative and 68.0–90.1 in the postoperative patients; *p* = 0.003. **c** Changes in the BI scores in patients incapable of ambulation within 7 days. The 95% C.I. was − 3.35 to 63.4 in the preoperative and − 13.2 to 54.9 in the postoperative patients; *p* = 0.33. **d** Change in the BI scores in patients AWD. The 95% C.I. was 21.9–76.2 in the preoperative and 51.3–91.5 in the postoperative patients; *p* = 0.15. **e** Change in the BI scores in patients DOD. The 95% C.I. was 15.4–52.8 in the preoperative and 41.5–83.5 in the postoperative patients; *p* = 0.11. **f** Changes in the BI scores in patients who died of disease within 3 months of surgery. The 95% C.I. was − 1.54 to 41.5 in the preoperative and − 33.0 to 68.0 in the postoperative patients; *p* = 0.31. **g** Changes in BI scores in patients who died of disease after more than 3 months. The 95% C.I. was 26.7–60.6 in the preoperative and 61.9–87.2 in the postoperative patients; *p* = 0.006

Figure 1 presents the changes of the BI scores after surgery. BI score comparisons before and after surgery in all patients showed statistically significant increments, suggesting improvement of ADL (Fig. 1a, *p* = 0.017, power = 0.97, effect size = 0.8). Figure 1b shows the changes of the BI scores in patients capable of ambulation within 7 days, which also suggested significant improvement of ADL (*p* = 0.003, power = 0.92, effect size = 0.8). However, we were unable to evaluate those in patients incapable of ambulation within 7 days because statistically underpowered (Fig. 1c). Changes of the BI scores in patients alive with disease (AWD) are shown in Fig. 1d, and those in patients dead of disease (DOD) are shown in Fig. 1e. While patients AWD were statistically underpowered, patients DOD showed no statistical differences in the BI scores after surgery (*p* = 0.11, power = 0.83, effect size = 0.8). Figure 1f and g present changes of the BI scores in patients dead within 3 months after surgery and patients alive 3 months after surgery, respectively. The latter presented significant improvement of ADL (*p* = 0.006, power = 0.96, effect size = 0.8). Data on patients who died within 3 months were statistically underpowered.

### Association between BI score gain and variables including tumor progression, ESCC grade, SINs, and radiation therapy status

Univariate logistic analysis was performed to identify the predictor variables associated with the BI score gains. However, no statistical significance was shown with tumor progression (*p* = 0.136), ESCC grades (*p* = 0.529), SINs (correlation coefficient = 0.294, *p* = 0.136), preoperative ambulation status, or radiation therapy status (*p* = 0.519).

## Discussion

Previously, Uei et al. reported 40 patients with spinal metastasis treated with MISt. They focused on the surgical technique, comparing prognosis with and without posterior decompression [30]. They concluded that MISt without decompression is advantageous for certain patients; however, they did not perform statistical analysis of the Barthel index (BI) scores between before and after surgery. Kwan et al. reported 50 cases of spinal metastasis treated with MISt [31]. They performed survival analysis after MISt and concluded that MISt is acceptable as a palliative surgery. From the view of impairment-driven cancer rehabilitation, we believe that MISt-related studies should be much more QOL-oriented. In this study, we classified the impairments of patients through our multidisciplinary tumor board. We identified patients whose main impairments were spinal (skeletal) instability and those who underwent MISt and received immediate rehabilitation. In order to control tumor growth, additional chemotherapy and/or radiotherapy are required. Ambulation status has considerably improved after the surgery. Although many presented with high degrees of cord compression before surgery, early ambulation was achieved in almost all patients, suggesting that metastatic paraplegia can be prevented even if skeletal instability has caused critical physical impairment to the patient (Table 2).

Skeletal stabilization through MISt using PPS may affect neurological recovery. In our series, neurological deficiencies were improved or maintained in 22 patients, but worsened in 5 patients. Tumor volume leading to spinal compression is dependent on tumor growth speed. However, in the cases in which tumor growth was relatively slow or controlled by adjuvant therapy, stabilization surgery decreased swelling of the spinal cord and hence skeletal pain, eventually supporting neurological recovery (Table 3).

Results from the comparison of BI scores before and after surgery may suggest a more detailed surgical indication. Although this study is a retrospective review with limited number of cases, sufficient statistical power supported the efficacy of MISt in the improvement of ADL in patients with neoplastic spinal cord compression. Furthermore, through subgroup analyses, we found good functional prognosis in patients capable of ambulation within 7 days, and in patients who could survive longer than 3 months after the surgery. In the former comparison, patients whose general conditions worsened because of impairments from underlying diseases unrelated to spine could not ambulate within 7 days even with a stabilized spine and eventually showed no improvement of ADL. From the latter, we postulate that a 3-month life expectancy is a possible indication for MISt with neoplastic spinal cord compression. These findings may indicate a population who can be treated using this approach.

According to the Silver’s model [11], rehabilitation should be offered only after their impairments have been identified and treated optimally, and all safety precautions and contraindications have been identified. For patients with neoplastic spinal cord compression, at first, the impairment should be clarified to confirm if MISt is effective. After the treatment, immediate rehabilitation can be provided to the patients to obtain early ambulation.

**Fig. 2:**
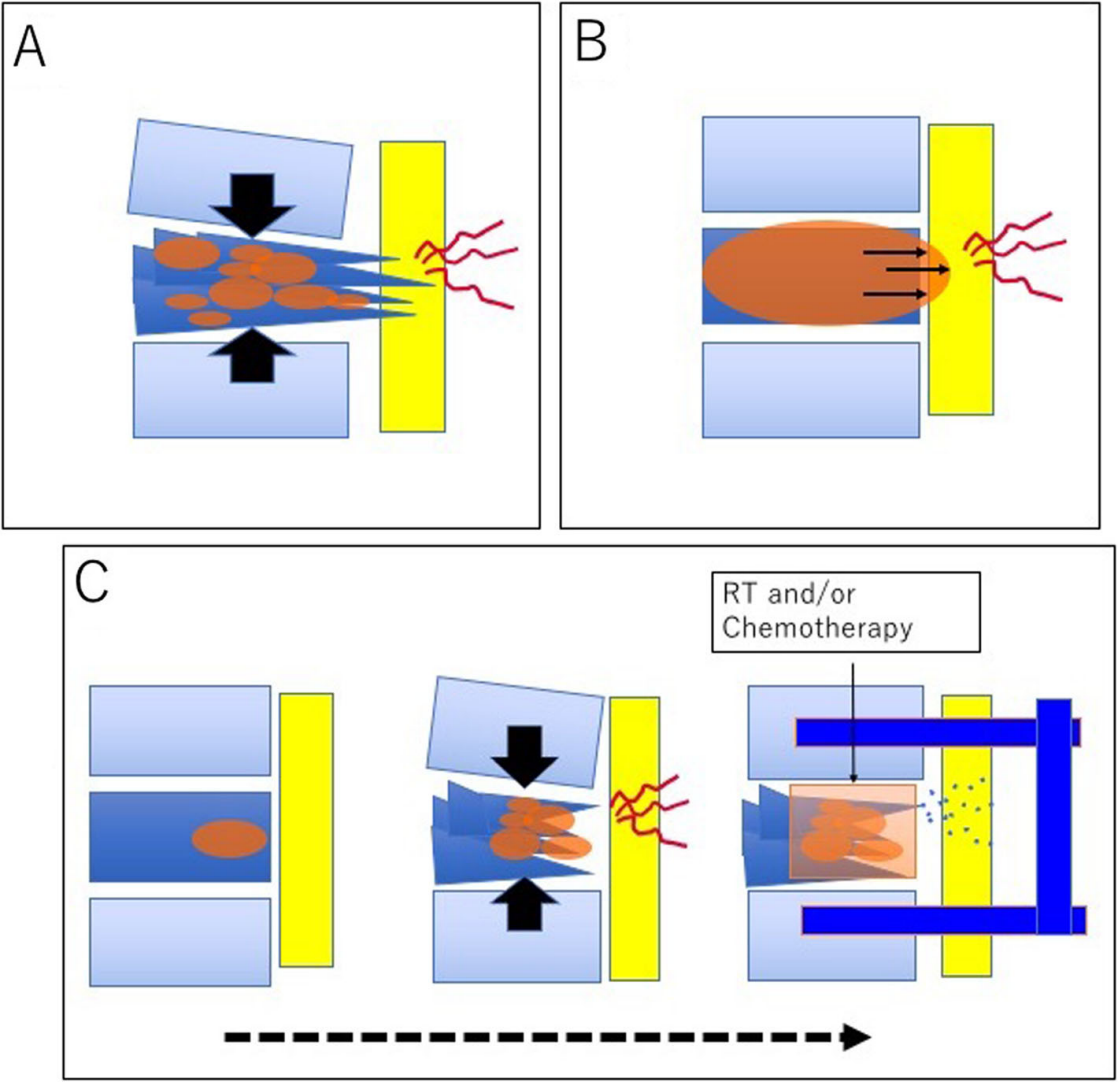
Schema of malignant spinal compression. Two representative statuses of malignant spinal compression are shown in **a** and **b**. **a** Skeletal instability as the major factor for impairment. **b** Tumor growth as the major factor for impairment. In the former case, spine stabilization may avoid the disability while in the latter, chemotherapy and/or radiotherapy are required (**c**)

To avoid preventable disabilities from metastatic paraplegia, spine-related impairments should be distinguished from other oncological impairments and further characterized based on two independent biological aspects, skeletal instability and tumor growth, as were done through the MDTB of our hospital. Two types of schemes of malignant spinal compression are shown in Fig. 2. Figure 2a shows a case with skeletal instability as the major factor for impairment, while Fig. 2b shows a case with tumor growth as the major factor for impairment. In terms of effective interventions to avoid disability, the former is an indication for spinal stabilization surgery, while the latter is an indication for additional chemotherapy and/or radiotherapy, typically in patients with treatment-sensitive tumors. However, our study involved a population of patients whose BI scores did not improve after surgery. Further investigations are required to estimate the possible progress of impairments due to underlying conditions unrelated to the spine.

There were several limitations in this study. This was a one arm retrospective study with a small sample size. The primary tumors were heterogeneous. Further larger-scale studies are required.

## Conclusion

In patients with neoplastic spinal cord compression, impairment-identification is of utmost importance. First of all, spine-specific impairments should be distinguished from other oncological impairments. These spine-specific impairments should then be evaluated based on two aspects: skeletal instability and tumor growth. For patients whose primary impairment was skeletal instability, MISt enabled them to ambulate early by immediate rehabilitation; this was reflected by their improved BI scores. Disability from neoplastic spinal compression can be prevented with appropriate interventions in those patients, which may increase the quality of life even if the patients are at the palliative stage of care.

## Data Availability

All date used and analyzed during this study are available from the corresponding author on reasonable request.

## Abbreviations

ADL: Activities of daily living
AWD: Alive with disease
BI: Barthel index
CSF: Cerebrospinal fluid
DOD: Dead of disease
ESCC: Epidural spinal cord compression
ICF: International Classification of Functioning, Disability, and Health
ICIDH: International Classification of Impairments, Disabilities, and Handicaps
MDTB: The multidisciplinary tumor board
MISt: Minimally invasive spine stabilization
PPS: Percutaneous pedicle screws
SINS: Spinal instability neoplastic scale

## References

[1] Surveillance Epidemiology and End Results. SEERCancer statistics review 1975–2014. Available at https://seer.cancer.gov/archive/csr/1975_2014/browse_csr.php?sectionSEL=2&pageSEL=sect_02_table.08, accessed June 30, 2020.

[2] Ito Y, Miyashiro I, Ito H, Hosono S, Chihara D, Nakata-Yamada K (2014). Long-term survival and conditional survival of cancer patients in Japan using population-based cancer registry data. The J-CANSIS Research Group. Cancer Science.

[3] Hewitt M, Rowland JH, Yancik R (2003). Cancer survivors in the United States: age, health, and disability. J Gerontol A Biol Sci Med Sci.

[4] Harrington CB, Hansen JA, Moskowitz M (2010). It’s not over when it’s over: long-term symptoms in cancer survivors—a systematic review. Int J Psychiatry Med.

[5] World Health Organization. International classification of impairments, disabilities, and handicaps: a manual of classification relating to the consequences of disease, published in accordance with resolution WHA29.35 of the Twenty-ninth World Health Assembly. World Health Organization. May 1976:1980 Available at https://apps.who.int/iris/handle/10665/41003.

[6] International Classification of Functioning, Disability and Health. Available at https://apps.who.int/iris/handle/10665/42407, accessed June 30, 2020.

[7] Stout NL, Alfano CM, Belter CW, Nitkin R, Cernich A, Lohmann SK (2018). A bibliometric analysis of the landscape of cancer rehabilitation research (1992-2016). J Natl Cancer Inst..

[8] Maltser S, Cristian A, Silver JK, Morris GS, Stout NL. A focused review of safety considerations in cancer rehabilitation. PM R. 2017;9(9S2):S415-28. 10.1016/j.pmrj.2017.08.403 PMC5627359 28942913

[9] Smith SR, Zheng JY (2017). The intersection of oncology prognosis and cancer rehabilitation. Curr Phys Med Rehabil Rep..

[10] Stubblefield MD, Hubbard G, Cheville A, Koch U, Schmitz KH, Dalton SO (2013). Current perspectives and emerging issues on cancer rehabilitation. Cancer..

[11] Silver JK, Baima J, Mayer RS (2013). Impairment-driven cancer rehabilitation: an essential component of quality care and survivorship. CA Cancer J Clin..

[12] McKinley WO, Huang ME, Brunsvold KT (1999). Neoplastic versus traumatic spinal cord injury: an outcome comparison after inpatient rehabilitation. Arch Phys Med Rehabil..

[13] Fattal C, Fabbro M, Gelis A, Bauchet L (2011). Metastatic paraplegia and vital prognosis: perspectives and limitations for rehabilitation care. Part 1. Arch Phys Med Rehabil..

[14] Ruppert LM (2017). Malignant spinal cord compression: adapting conventional rehabilitation approaches. Phys Med Rehabil Clin N Am..

[15] Fisher CG, DiPaola CP, Ryken TC, Bilsky MH, Shaffrey CI, Berven SH (2010). A novel classification system for spinal instability in neoplastic disease: an evidence-based approach and expert consensus from the Spine Oncology Study Group. Spine (Phila Pa 1976)..

[16] Bilsky MH, Laufer I, Fourney DR, Groff M, Schmidt MH, Varga PP (2010). Reliability analysis of the epidural spinal cord compression scale. J Neurosurg Spine..

[17] Tomita K, Kawahara N, Mizuno K, Toribatake Y, Kim SS, Baba H, Rao RS, Deo MG, Sanghri LD, Mittra I (1994). Total en bloc spondylectomy for primary malignant vertebral tumors. Proceedings of the 16th International Cancer Congress.

[18] Tomita K, Kawahara N, Baba H, Tsuchiya H, Fujita T, Toribatake Y. Total en bloc spondylectomy. A new surgical technique for primary malignant vertebral tumors. Spine (Phila Pa 1976). 1997;22(3):324-33. 10.1097/00007632-199702010-00018 9051895

[19] Singh K, Samartzis D, Vaccaro AR, Andersson GB, An HS, Heller JG (2006). Current concepts in the management of metastatic spinal disease. The role of minimally-invasive approaches. J Bone Joint Surg Br..

[20] Schwab JH, Gasbarrini A, Cappuccio M, Boriani L, De IF, Colangeli S, et al. Minimally invasive posterior stabilization improved ambulation and pain scores in patients with plasmacytomas and/or metastases of the spine. Int J Surg Oncol. 2011. 10.1155/2011/239230. 10.1155/2011/239230 PMC3263662 22312498

[21] Tancioni F, Navarria P, Pessina F, Marcheselli S, Rognone E, Mancosu P, et al. Early surgical experience with minimally invasive percutaneous approach for patients with metastatic epidural spinal cord compression (MESCC) to poor prognoses. Ann Surg Oncol. 2012. 10.1245/s10434-011-1894-x. 10.1245/s10434-011-1894-x 21743979

[22] Rao PJ, Thayaparan GK, Fairhall JM, Mobbs RJ (2014). Minimally invasive percutaneous fixation techniques for metastatic spinal disease. Orthop Surg..

[23] Mahoney FI, Barthel DW (1965). Functional evaluation: the Barthel index. Md State Med J..

[24] Tokuhashi Y, Matsuzaki H, Oda H, Oshima M, Ryu J (2005). A revised scoring system for preoperative evaluation of metastatic spine tumor prognosis. Spine (Phila Pa 1976)..

[25] Katagiri H, Okada R, Takagi T, Takahashi M, Murata H, Harada H (2014). New prognostic factors and scoring system for patients with skeletal metastasis. Cancer Med..

[26] Frankel HL, Hancock DO, Hyslop G, Melzak J, Michaelis LS, Ungar GH (1969). The value of postural reduction in the initial management of closed injuries of the spine with paraplegia and tetraplegia. Paraplegia.

[27] Rollnik JD. The Early Rehabilitation Barthel Index (ERBI) rehabilitation (Stuttg). 2011;50(6):408-11. 10.1055/s-0031-1273728 21626475

[28] Castiglia SF, Galeoto G, Lauta A, Palumbo A, Tirinelli F, Viselli F (2017). The culturally adapted Italian Version of the Barthel Index (IcaBI): assessment of structural validity, inter-rater reliability and responsiveness to clinically relevant improvements in patients admitted to inpatient rehabilitation centers. Funct Neurol..

[29] Hobart A, Thompson A (2001). The five items Barthel index. J Neurol Neurosurg Psychiatry..

[30] Uei H, Tokuhashi Y, Maseda M, Nakahashi M, Sawada H, Nakayama E (2018). Comparison between minimally invasive spine stabilization with and without posterior decompression for the management of spinal metastases: a retrospective cohort study. J Orthop Surg Res..

[31] Kwan MK, Lee CK, Chan CY (2016). Minimally invasive spinal stabilization using fluoroscopic-guided percutaneous screws as a form of palliative surgery in patients with spinal metastasis. Asian Spine J..

